# A Case Report and Literature Review of the Outcome of Linezolid-Induced Optic and Peripheral Neuropathy in Patients With Multidrug-Resistant Pulmonary TB

**DOI:** 10.3389/fneur.2022.908584

**Published:** 2022-06-24

**Authors:** Safia Bano, Ahmad Nawaz, Ahsan Numan, Muhammad Aarish Hassan, Muhammad Bilal Ahmad Shafique

**Affiliations:** King Edward Medical University, Lahore, Pakistan

**Keywords:** case report, drug-resistant tuberculosis, linezolid (LZD), optic neuropathy, peripheral neuropathy

## Abstract

Linezolid is a second-line medication used to treat tuberculosis that has become resistant to multiple drugs. Linezolid has been shown to be effective in treating drug-resistant TB. However, long-term therapy is hampered by the related side effects, such as ocular and peripheral neuropathy. We recently encountered a 32-year-old male undergoing linezolid therapy for 12 months for multidrug-resistant tuberculosis who presented with progressive painless visual impairment and peripheral neuropathy symptoms in lower limbs as well as ataxic gait. Nerve conduction study findings of length-dependent axonal sensory polyneuropathy with bilateral optic neuropathy evident on fundoscopy suggested a case of toxic neuropathy. Following the termination of linezolid, follow-up visits revealed an improvement in visual symptoms. While there has been no discernible improvement or deterioration of peripheral neuropathy. In a developing country like Pakistan, where the rising number of cases of multidrug-resistant tuberculosis and its management is a major problem, physicians should be made aware of linezolid induced neuropathy so that close follow-up sessions for patients on long-term linezolid therapy can be arranged to avoid serious neurological consequences.

## Introduction

Tuberculosis is a communicable disease that accounts for a high burden of global mortality and morbidity. Tuberculosis (TB) affects 10 million individuals every year throughout the world. The global incidence of MDR-TB is 3.4% in new cases and 18% in previously treated cases (1). The emergence of resistance to first-line classical anti-tuberculous therapy is frequent in developing and economically poor countries. Therefore, second-line anti-tuberculosis agents are utilized frequently for the treatment of extensively drug-resistant (XDR) and multidrug-resistant tuberculosis (MDR)tuberculosis. Linezolid, a synthetic oxazolidinone antimicrobial, is one of the second-line drugs approved by the world health organization for the treatment of multidrug-resistant Mycobacterium tuberculosis (2, 3). Although Linezolid showed promised efficacy its long-term use has been associated with serious adverse effects such as myelosuppression, as well as optic and peripheral neuropathies, which have been a particular concern for patients with drug-resistant tuberculosis, who need prolonged therapy (4–6). According to the evidence, the most relevant risk factor for linezolid-induced neurotoxicity, rather than the dose or indication for therapy, is extended use. The safety of linezolid treatment for up to 28 days has been established. Long-term use outside the 28-day window has been linked to the optic and peripheral neuropathies. Patients who have been taking linezolid for more than 28 days should be checked for signs of peripheral and optic neuropathy (7). Recently, we encountered a case of a 32-year-old male treated with the linezolid-based regime for multidrug-resistant tuberculosis who presented with the chief complaints of progressive deterioration of vision and Signs and symptoms suggestive of peripheral neuropathy. The identification of the culprit drug and its cessation resulted in the improvement of visual acuity and color perception of the patient and halted the progression of peripheral neuropathy.

## Case Presentation

**Figure 1:**
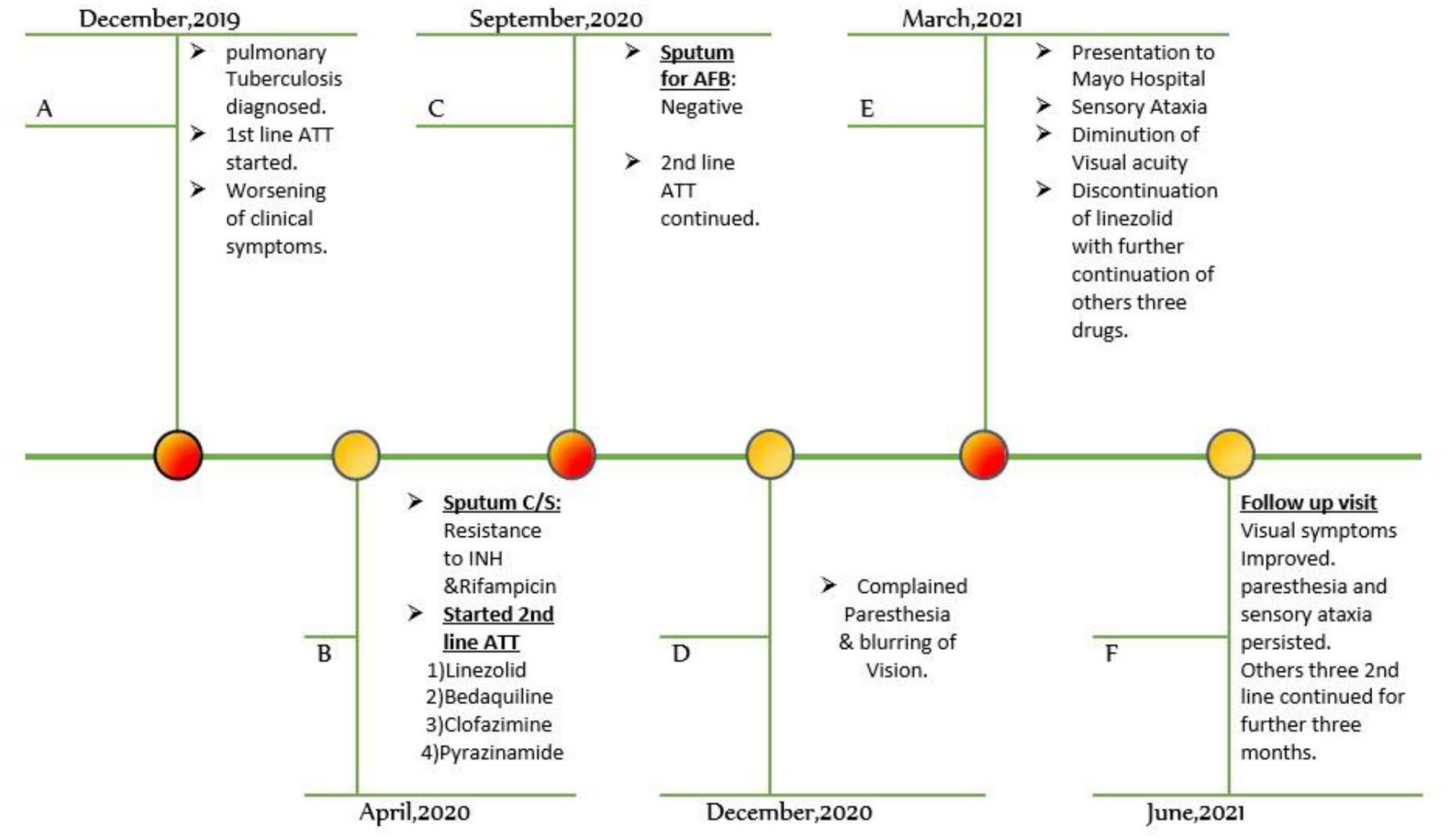
Timeline of events. ATT, anti-tuberculous treatment, AFB. acid-fast bacilli, C/S, culture and sensitivity, INH, isoniazid.

A 32-year-old Pakistani man was admitted to the neurology department of a tertiary care hospital with a four-month history of numbness and severe tingling sensations in his hands and feet, which later progressed within 2 months to his forearms and legs in a glove and stocking manner. The patient also complained of unsteady gait due to loss of balance. He also complained of a rapid, painless diminution of vision in both eyes for 4 months. He was normotensive and non-diabetic with no history of smoking or alcohol intake and had normal bowel habits. On inquiry into his dietary pattern, he used to eat meat 2–3 times per week. Previously, he had no history of neuropathy, nephropathy, retinopathy, or macrovascular complications. He had no history of joint pain or any features favoring autoimmune disease, vasculitis, or thyroid-related disorders. He reported no recent exposure to chemicals. He belonged to a lower-middle socioeconomic family and none of his close family members suffered from such complaints. He was not taking any medications besides a linezolid-based regimen for MDR tuberculosis. He was diagnosed with pulmonary tuberculosis in December 2019 and classical first line ATT was initiated. Despite his good compliance and adherence to treatment, his clinical symptoms of tuberculosis infection worsened. The patient developed resistance to Isoniazid and Rifampicin after 4 months of classical first line ATT, which was confirmed by sputum for culture and drug sensitivity. After that, he was diagnosed with multidrug-resistant pulmonary tuberculosis, and a Linezolid-based regimen including Clofazimine, Bedaquiline, and Pyrazinamide was instituted. Linezolid was initiated orally at a dose of 600 mg. His sputum for AFB turned negative after 6 months of 2nd line antituberculosis drug treatment (Figure 1). The treatment regime was continued further as per WHO guidelines.

## Physical Examination

On general physical examination, the patient was afebrile with normal vital signs. An ophthalmological examination revealed a significant diminution of visual acuity in both eyes limited to hand motion, finger counting, and light perception. Contrast sensitivity and color perception were reduced in both eyes. However, the pupils were normal, equal in size, and they were reactive to light equally. Fundus evaluation revealed bilaterally symmetrically edematous discs. Neurological examination of the motor system revealed normal power (MRC scale) grade 4+/5 in all limbs proximally and distally. Sensations of pinprick, fine touch and proprioception were impaired in lower limbs up to the knee. His gate was a broad-based ataxic gait with a positive Romberg's sign. The remainder of the systemic examination was unremarkable.

## Investigations

**Figure 2:**
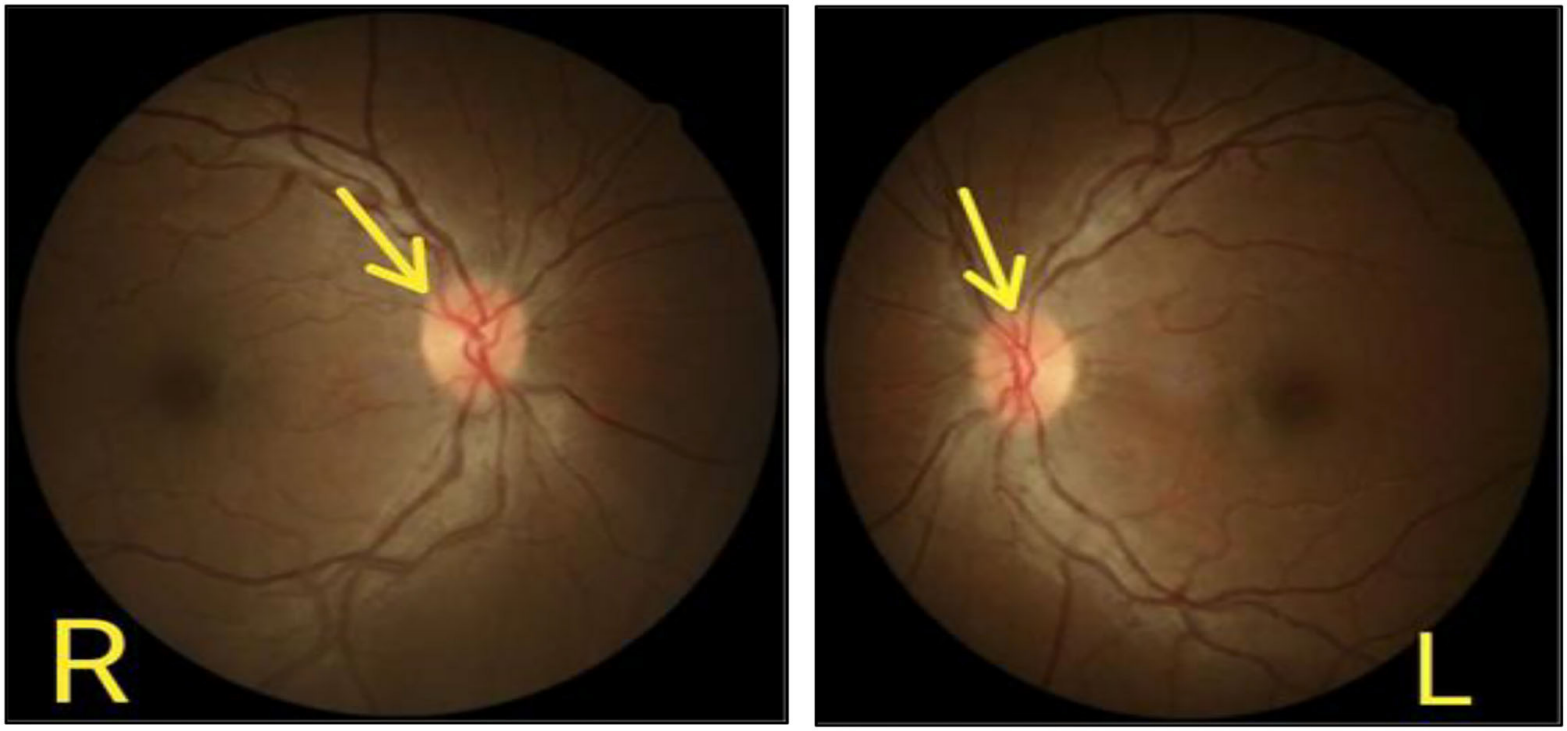
Optic disc edema of right and left eye.

A complete blood picture showed mild normocytic normochromic anemia. Liver enzymes, serum Urea and creatinine, serum electrolytes, thyroid profile, serum ferritin, serum vitamin B12, hemoglobin A1c, anti-neutrophilic antibody titers, and Serum protein electrophoresis levels were within the normal reference range. Cerebrospinal fluid analysis showed a minimal rise in protein (52 mg/dl) with normal CSF glucose and normal white blood cells and differential counts. The cerebrospinal fluid's opening pressure was 15 cm2H20. Nerve conduction studies showed a marked reduction in the amplitude of sensory nerves of the lower limbs compared to the upper limbs. Nerve conduction study findings were suggestive of length-dependent axonal sensory polyneuropathy (Table 1). Optical coherence tomography imaging of the optic nerve head revealed the increased thickness of retinal nerve fiber layers of 320 and 330 μm in the right and left eyes, respectively (normal 220+20 um). Fundoscopic images revealed bilateral optic disc edema (Figure 2). A visual field test performed by a visual field Analyzer revealed bilateral central scotoma Magnetic resonance imaging of the brain and optic nerve was unremarkable. Due to a lack of appropriate testing in Pakistan, the concentration of linezolid in the blood could not be determined.

Table 1Showing Nerve conductive studies of tested nerves with the interpretation of results and final conclusion (abnormal parameters are shown in bold).

**Table:** 

**Motor nerves**	**DML/AMP**	**PML/AMP**	**Distance**	**Velocity**	**F-waves**
Right Median	3.0 (11.5 mv)	7.1 (11.2 mv)	26 cm	63 m/sec	25 m/sec
Left Median	2.1(11.9 mv)	6.2 (11.6 mv)	26 cm	66 m/sec	26 m/sec
Right Ulnar	3.1 (13.5 mv)	7.2 (11.2 mv)	27 cm	66 m/sec	26 m/sec
Left Ulnar	2.2 (11.8 mv)	6.8 (10 mv)	27 cm	59 m/sec	27 m/sec
Right Common peroneal	4.4 (4.4 mv)	12.9 (3.7 mv)	35 cm	41 m/sec	44 m/sec
Left Common peroneal	5.5(3.9 mv)	14.9(3.3mv)	35 cm	45 m/sec	45 m/sec
Right Tibial	4.0 (15 mv)	12.9 (11 mv)	40 cm	45 m/sec	50 m/sec
Left Tibial	4.8 (15 mv)	12.9 (9 mv)	40 cm	49 m/sec	49 m/sec

**Table:** 

**Sensory Nerves**	**Latency**	**Amplitude**	**Distance**	**Conduction velocity**
Right Median	2.9 m/sec	**5.3 uv**	15 cm	52 m/sec
Left median	2.9 m/sec	**6.0 uv**	15 cm	52 m/sec
Right Ulnar	2.3 m/sec	**4.8 uv**	13 cm	56 m/sec
Left Ulnar	2.2 m/sec	**4.0 uv**	13 cm	59 m/sec
Right Sural	2.2 m/sec	**4.0 uv**	14 cm	48 m/sec
Left Sural	2.5 m/sec	**5.0 uv**	14 cm	48 m/sec
Right superficial peroneal	**No response**	**No response**	**No response**	**No response**
Right superficial peroneal	**No response**	**No response**	**No response**	**No response**
**Report Interpretation:** I) Motor nerve conduction studies showed a normal study of the tested nerves in both upper and lower limbs. II) Sensory nerve conduction study reveals reduced sensory nerve action potential to tested nerves of both upper limbs and lower limbs. III) Absent response of bilateral superficial peroneal nerves.				
**Conclusion**: A nerve conduction study reveals sensory neuropathy more severely affects the lower limbs				

*DML, distal motor latency; PML, proximal motor latency; AMP, amplitude*.

## Differential Diagnosis

Based on the clinical history, physical examination results, and laboratory testing, we made the tentative diagnosis of metabolic neuropathy. While there was no significant family history of peripheral neuropathy, inherited causes of neuropathy were ruled out. Normal eating habits and a healthy lifestyle ruled out nutritional neuropathy. Alcohol-induced neuropathy was also excluded due to the absence of a history of drinking and the subacute development of symptoms. Normal test findings for common metabolic neuropathy causes rule out all the other common metabolic neuropathy causes. Because there was no prior evidence of reduced visual acuity or peripheral neuropathy symptoms, we concluded that toxic neuropathy was the cause of the event. The study of the literature found no evidence of clofazimine, pyrazinamide, or Bedaquiline-induced neuropathy. As a result, we were able to establish a clinical diagnosis of linezolid-induced ocular and peripheral neuropathy. Due to a lack of resources, the serum concentration of linezolid could not be determined, which would have strengthened our diagnosis.

## Treatment

After 6 months of 2nd line ATT, sputum for AFB was found to be negative. Linezolid was promptly stopped from the treatment plan after consultation with the Chest-infections and Tuberculosis unit since the diagnosis of linezolid produced neuropathy was confirmed. As continuing to use the offender may aggravate the toxic symptoms or result in additional negative consequences. While Pyrazinamide, Bedaquiline, and Clofazimine were given for another 3 months. The overall treatment time was around 18 months, with linezolid treatment lasting about 12 months. For 12 weeks, 75 mg of oral Gabapentin was added to the treatment regimen for symptomatic alleviation of peripheral neuropathy symptoms.

## Outcome and Follow-up

At 3 months after the discontinuation of the drug, the Patent experienced a subjective improvement in vision, color perception, and paresthesia in the lower limbs. His best-corrected visual acuity (BRVA)checked with Snellen's chart was improved to 20/50 in both eyes. Color vision tested with ischara color charts was normal. On the neurological assessment of the sensory system, pinprick, light touch sensations, and proprioception was not improved in lower limbs with no improvement in gait disturbances. Findings on repeat nerve conduction studies remained unchanged and no improvement or further worsening was seen in the amplitude of tested sensory nerves. To date, his gait is still ataxic with no objective improvement in peripheral neuropathic symptoms. AFB remained undetected on sputum smear even after the discontinuation of linezolid and on completion of therapy with the other three drugs. During and after completing the Antituberculosis treatment, neither any other adverse effect nor hepatic, renal, or hematological abnormalities were seen.

## Discussion

Linezolid, an oxazolidinone antibiotic, exhibits wide antibacterial efficacy against Gram-positive pathogens such as Methicillin and vancomycin-resistant Staphylococcus aureus (MRSA, VRSA), Vancomycin-resistant Enterococcus species (VRE), and drug-resistant Mycobacterium tuberculosis (DR-TB) (13). Linezolid is a bacteriostatic antibiotic that inhibits bacterial ribosomal protein synthesis by binding to the bacterial 23S ribosomal RNA of the 50S subunit, preventing the development of the 70S RNA initiation complex. It also prevents bacterial pathogens from releasing toxins (14). A thorough review of the literature found that linezolid had a success rate of more than 80% in treating multidrug-resistant Mycobacterium TB (15, 16). In our situation, the sputum for AFB was negative after 6 days.

Linezolid is a drug that is well-tolerated and generally safe. However, the side effects limit its long-term usage in multidrug-resistant tuberculosis. Linezolid-induced toxic neuropathy is always characterized by bilateral painless progressive visual loss and color vision impairment. Bilateral central and cecocentral scotomas are observed in the visual field. Fundoscopy examination reveals temporal pallor, bilateral symmetrical disc edema, or essentially normal (Table 2). In the case of past ophthalmological abnormalities such as amblyopia or exenteration of either eye, the finding can be asymmetrical. With nerve conduction studies consistent with length-dependent axonal sensory polyneuropathy, peripheral neuropathy might appear clinically as numbness, paresthesia, and impairment in pain, temperature, and light touch sensations or loss of proprioception in a glove and stocking distribution.

**Table 2:** Illustrates the dose and duration of linezolid exposure, as well as the outcomes in cases reported from 2021 to 2014.

**References**	**Indication /Systemic illness**	**Dose of linezolid** **(mg)**	**Duration of treatment before symptoms (months)**	**Symptoms at the time of onset**	**VA/Visual field /Fundus findings at diagnosis**	**Peripheral Neuropathy /NCS findings at diagnosis**	**Outcome**
Aljebreen et al., (8)	XDR-TB	600 mg/B. d	5 months	Bilateral visual impairment	1)VA 20/200 in both eyes 2) Poor color vision 3) ceco-central scotomas in both eyes 4) Bilateral blurred disc margins	Not reported	**Three months** after cessation, VA was 20/25 in the right eye and 20/28 in the left eye. Color vision was restored in both eyes Fundus showed mild temporal disk pallor, more prominent in the left eye. Visual field testing was unremarkable.
Agashe and Doshi, (9)	XDR-TB	Not reported	12 months	Bilateral Painless Blurring of Vision	1) VA limited to Finger counting at two meters in both eyes 2)Poor color vision 3) Bilateral Disc oedema	Not Reported	Normal visual acuity And Disc edema completely resolved at **3**^**rd**^ **month** of cessation of drug
Lee et al., (10)	MDR-TB	600 mg/O. d	6 months	1) Bilateral Painless Blurring of Vision 2) Paresthesia in distal extremities	1) VA:20/300 in right eye and 20/200 in left eye 2)Poor color vison 3) bilateral central scotoma 4) Bilateral mild Disc oedema	Paresthesia in distal extremities	Normal visual acuity, Normal visual field And Disc edema completely resolved at **3rd month** of cessation of drug. Paresthesia of the feet did not fully recover
Shah et al., (11)	XDR-TB	600 mg/O. d	14 months	Painless Blurred Vision	1)VA: 6/60 in both eyes 2)Poor color vison 3) bilateral central scotoma 4) Bilateral Disc oedema	Not Reported	Normal visual acuity, Normal color vision, Decreased size of central scotoma, Disc edema completely resolved at **1.5 month** of cessation of drug.
Swaminathan et al., (12)	MDR-TB/DM	600 mg/O. d	8 months	Not reported	Normal examination	Paresthesia and mild intermittent pain in feet NCS: Sensory and motor neuropathy of axonal type	clinical improvement of Paresthesia and pain in feet at **21- months** cessation of linezolid
Karuppannasamy et al., (4)	XDR-TB	600 mg/O. d	6 months	Painless diminution of vision of both eyes	1) VA:20/200 in both eyes 2) Poor color vision 3) Peripheral constriction and supertemporal quadrantanopia in right eye. Quadrantanopia in left eye 4) Bilateral Disc oedema		Follow up visit after one **month** of discontinuation of drug showed Normal visual acuity in both eyes Optic disc edema resolved Visual field showed partial improvement with residual quadrantanopia

*XDR, Extended drug- resistant; MDR, Multidrug-resistant; TB, Tuberculosis; VA, Visual acuity; O.d, once daily; B.d, twice a day*.

The toxicity of the medicine is most likely related to the length of treatment. It is relatively safe when used for a period of fewer than 28 days (16). In the majority of reported cases of neuropathy, the dose was 600 mg once daily, and the length of exposure ranged from 5 to 14 months (8–11). After conducting a systematic review and meta-analysis of the efficacy and safety of therapy with linezolid-containing regimens in the treatment of drug-resistant tuberculosis, Zhang X et al. determined that neurotoxicity with peripheral neuropathy occurs in 30% of patients who had received doses < or equal to 600 mg/day for 4–6 months (7, 16). In our case, the patient was given a dose of 600 mg/day for 9 months prior to the onset of symptoms.

Although the specific cause of its toxicity is uncertain, Linezolid is thought to affect the visual nerve by interfering with oxidative phosphorylation in the mitochondria. The majority of the energy during conduction is generated by mitochondria via oxidative phosphorylation, which requires electron transport along a convoluted chain. Reactive oxygen species are formed when electron transport fails. The combination of energy shortage and oxidative stress results in cytochrome c leaking from the mitochondrial pore, which causes apoptosis and nerve damage. When functional impairment occurs without axon loss, the damage can be reversed. When the medication is withdrawn, the majority of people with optic nerve injury improve, likely because the triggering factor is removed before apoptosis and permanent axonal loss occurs (17, 18).

Linezolid is non-enzymatically metabolized in the liver without involving the CYP450 oxidative system. Nonrenal clearance accounts for 65% of a drug's clearance, with only 30% of the remaining dose excreted unchanged in the urine. Because of linezolid's unique pharmacologic and pharmacokinetic features, there is no need to change the recommended dose in mild to severe renal or hepatic failure (19). In our situation, the prolonged period without regular monitoring for adverse effects was a likely contributing factor to the neurotoxicity. Furthermore, renal and liver function tests were within normal limits, and the patient was taking the low dose linezolid 600 mg orally once daily. The occurrence and risk factors for linezolid poisoning have not been thoroughly investigated. Age, gender, and mitochondrial DNA polymorphisms can be the possible risk factors for linezolid poisoning. Several researchers hypothesized that patients with pre-existing neurologic sequelae or risk factors, such as alcoholism, diabetes, or concurrent use of chemotherapeutic medicines and/or antiretroviral therapy, were more susceptible to linezolid-induced neurotoxicity (5).

Swaminathan et al. described a diabetic child who had developed peripheral neuropathy while on long-term linezolid therapy for tuberculosis. Optic neuropathy resolved in this patient 21 months after the drug was stopped. This case study shows how co-morbidities, such as diabetes, can increase the likelihood of developing unpleasant neuropathic symptoms and delay recovery. As a result, careful glycemic control and effective treatment of related comorbidities can reduce the severity of neurological sequelae while also improving the chances of TB recovery (12). It is still unclear whether linezolid serum concentrations, long-term exposure, or coexisting systemic illness can predict adverse effects.

There is no known treatment for linezolid-induced neurotoxicity. In patients who develop neuropathic symptoms, linezolid should be stopped immediately. Linezolid-induced optic neuropathy is nearly reversible, but the exact duration of symptom resolution following drug discontinuation is unknown. The majority of reported cases of optic neuropathy resolved within 1–3 months of drug discontinuation (8–11). However, the presence of other systemic illnesses can impede optic neuropathy recovery. There has been no reported case of complete recovery of peripheral neuropathic symptoms. A review of the literature on a retrospective study of 16 patients on a linezolid-based regimen for MDR-tuberculosis found no improvement in sensory neuropathy in any of the patients (12).

Diagnosis of drug-induced toxicity is extremely challenging in countries where the facilities to measure the serum therapeutic concentration are not available. Here, we demonstrated a case of optic and peripheral neuropathy which developed 9 months after the linezolid base therapy. Previously insignificant history of any etiological factor causing neuropathy, findings of sensory axonal neuropathy on nerve conduction study and bilateral edematous optic disc on fundoscopy suggested toxic neuropathy. Improvement in symptoms after the discontinuation of the drug further strengthened the diagnosis of drug-induced toxic neuropathy. There are certain limitations that the authors would like to acknowledge that the serum concentration of the drug could not be carried out to confirm our diagnosis. However, reporting the educational value of our case for practicing physicians and ophthalmologists is of paramount value in highlighting the need to evaluate the visual acuity before starting the linezolid base regime and to arrange close follow-up sessions as often as possible for patients receiving long-term therapy with linezolid.

## Conclusion

We report a diagnostically extremely challenging case of drug-induced toxic neuropathy. The classic clinical signs and symptoms of optic disc swelling and peripheral neuropathy have largely fallen out of favor and that can be seen in most cases of linezolid base long-term therapy. Physicians need to monitor and recognize such symptoms clinically to stop the toxic drug early to prevent optic and peripheral neuropathy from worsening.

## Patient Perspective

I have been experiencing the severe disabling tingling sensations in my feet and was living with monochromic vision for about 12 months. I had consulted many physicians and ophthalmologists for my problem. But the root cause could not be figured out. After additional tests, Now I have been aware of cause of my disease and iam well aware of lack of definitive treatment for my gait disturbance. But iam still happy for this partial recovery and improvement in my vision and color perception.

## Data Availability Statement

The raw data supporting the conclusions of this article will be made available by the authors, without undue reservation.

## Ethics Statement

The studies involving human participants were reviewed and approved by Ethical Review Committee, Mayo Hospital Lahore. The patients/participants provided their written informed consent to participate in this study. Written informed consent was obtained from the individual(s) for the publication of any potentially identifiable images or data included in this article.

## Author Contributions

SB have made substantial contributions to the conception, design, and acquisition of data. ANa have been involved in drafting the manuscript or revising it critically for important intellectual content. ANu have given final approval to the version to be published. MH and MS have contributed to the analysis and interpretation of data. All authors contributed to the article and approved the submitted version.

## Conflict of Interest

The authors declare that the research was conducted in the absence of any commercial or financial relationships that could be construed as a potential conflict of interest.

## Publisher's Note

All claims expressed in this article are solely those of the authors and do not necessarily represent those of their affiliated organizations, or those of the publisher, the editors and the reviewers. Any product that may be evaluated in this article, or claim that may be made by its manufacturer, is not guaranteed or endorsed by the publisher.
